# Effect of Blood Transfusion on Cerebral Hemodynamics and Vascular Topology Described by Computational Fluid Dynamics in Sickle Cell Disease Patients

**DOI:** 10.3390/brainsci12101402

**Published:** 2022-10-18

**Authors:** Russell P. Sawyer, Sirjana Pun, Kristine A. Karkoska, Cherita A. Clendinen, Michael R. DeBaun, Ephraim Gutmark, Riccardo Barrile, Hyacinth I. Hyacinth

**Affiliations:** 1 Department of Neurology and Rehabilitation Medicine, University of Cincinnati College of Medicine, 231 Albert Sabin Way, Cincinnati, OH 45267-0525, USA; 2 Department of Biomedical Engineering, College of Engineering and Applied Science, University of Cincinnati, Cincinnati, OH 45219, USA; 3 Division of Hematology/Oncology, Department of Internal Medicine, University of Cincinnati College of Medicine, Cincinnati, OH 45219, USA; 4 Department of Psychology, Behavioral and Cognitive Neuroscience, University of Florida, Tampa, FL 33620, USA; 5 Vanderbilt-Meharry Center of Excellence in Sickle Cell Disease, Vanderbilt University Children’s Hospital, Nashville, TN 37232, USA; 6 Department of Aerospace Engineering and Engineering Mechanics, College of Engineering and Applied Science, University of Cincinnati, Cincinnati, OH 45219, USA

**Keywords:** sickle cell disease, stroke, neuroimaging, hematology, computational fluid dynamics

## Abstract

The main objective of this study was to demonstrate that computational fluid dynamics (CFD) modeling can be used to study the contribution of covert and overt vascular architecture to the risk for cerebrovascular disease in sickle cell disease (SCD) and to determine the mechanisms of response to therapy such as chronic red blood cell (cRBC) transfusions. We analyzed baseline (screening), pre-randomization and study exit magnetic resonance angiogram (MRA) images from 10 (5 each from the transfusion and observation arms) pediatric sickle SCD participants in the silent cerebral infarct transfusion (SIT) trial using CFD modeling. We reconstructed the intracranial portion of the internal carotid artery and branches and extracted the geometry using 3D Slicer. We cut specific portions of the large intracranial artery to include segments of the internal carotid, middle, anterior, and posterior cerebral arteries such that the vessel segment analyzed extended from the intracranial beginning of the internal carotid artery up to immediately after (~0.25 inches) the middle cerebral artery branching point. Cut models were imported into Ansys 2021R2/2022R1 and laminar and time-dependent flow simulation was performed. Change in time averaged mean velocity, wall shear stress, and vessel tortuosity were compared between the observation and cRBC arms. We did not observe a correlation between time averaged mean velocity (TAMV) and mean transcranial Doppler (TCD) velocity at study entry. There was also no difference in change in time average mean velocity, wall shear stress (WSS), and vessel tortuosity between the observation and cRBC transfusion arms. WSS and TAMV were abnormal for 2 (developed TIA) out of the 3 participants (one participant had silent cerebral infarctions) that developed neurovascular outcomes. CFD approaches allow for the evaluation of vascular topology and hemodynamics in SCD using MRA images. In this proof of principle study, we show that CFD could be a useful tool and we intend to carry out future studies with a larger sample to enable more robust conclusions.

## 1. Introduction

Sickle cell disease (SCD) is the most common inherited hemoglobinopathy worldwide, affecting over 300,000 live births each year [1]. SCD results from a base substitution at the sixth amino acid position in the β-globin chain [2], which causes red blood cells (RBCs) to “sickle” under hypoxic and/or acidotic conditions, which results in microvascular occlusion, infarction, and end organ damage. SCD causes significant morbidity and early mortality; neurovascular complications are particularly devastating and range from overt stroke (clinical stroke) to progressive cognitive decline even in the absence of neuroanatomical changes [3]. Indeed, 11% of untreated children with SCD will experience an overt ischemic stroke by age 20 years, while approximately 30% of individuals with SCD have evidence of silent cerebral infarctions (SCI), defined as areas of white matter hyperintensity seen on T2-weighted brain MRI [4,5]. These “silent” infarctions are not truly silent, as they are associated with worse performance on measures of cognitive function (using the proxy measure of full-scale intelligence quotient) compared to individuals with SCD without SCI [6].

The large-vessel disease of overt stroke in SCD has been well-characterized, with evidence of stenosis, downstream occlusion, and tortuosity of the affected vessels, predominantly within the Circle of Willis. These changes correlate with an elevated arterial blood flow velocity of greater than 200 cm/s (cm/s) in the anterior cerebral artery, middle cerebral artery, or internal carotid artery on transcranial Doppler ultrasound (TCD) [7,8,9]. SCI has been associated with intracranial large vessel stenosis, as well as extracranial internal carotid artery stenosis and significant anemia (baseline hemoglobin below 7 g/dL) [10]. Additionally, evidence from angiography and autopsy have documented several pathological changes in the cerebral macro- and microvasculature including stenosis and fibrosis, exuberant intimal growth and endothelial proliferation, and formation of sickle red cell sludge in small blood vessels as contributors to SCD-related cerebral vasculopathy [11,12,13,14].

Studies in subjects without SCD using endothelialized microfluidics devices have shown that computational fluid dynamics (CFD) models built from magnetic resonance angiography (MRA) images can be used to identify sections of a vessel or flow channel with uneven internal arterial surfaces that may induce regions of low wall shear stress, which are associated with greater endothelial activation and possibly intimal hyperplasia that may predispose to stenosis [15,16,17,18]. In a recent study with three patients (one healthy control and 2 with SCD), Rivera et al. applied CFD to the internal carotid artery (ICA) and its main branches, and demonstrated the presence of internal arterial wall surfaces with regions of low wall shear stress and more disturbances in blood flow; they hypothesized that these regions are predisposed to stenosis and possibly contribute to the observed higher TCD velocities and stroke risk in the children with SCD [19].

However, SCD patients without evidence of extra- and intracranial stenosis and normal TCD velocities still experience overt stroke and SCI [20,21,22]; further investigation into the molecular and structural mechanisms for these pathologies is warranted.

In this study, we used MRA images from children with SCD with baseline TCD velocities < 200 cm/s to create CFD models to characterize the topology and flow parameters of the left and right ICA and main branches in ten patients, five each from the observation and transfusion arms of the SIT trial. We hypothesized that velocity profile, wall shear stress, and vessel topology (tortuosity) are correlated and can be used as markers of progression of cerebrovascular disease and response to blood transfusion therapy in children with SCD, especially in the setting of TCD velocities < 200 cm/s.

- This study will represent the largest study to date to utilize MRI images from patients with SCD to model blood flow and wall shear stress in segments of the ICA and MCA.
- It will also be the first to perform this modeling in a longitudinal fashion, in children with normal TCD velocity at baseline thus allowing us to take the step towards determining whether the CFD measures could have predictive benefit.
- It is also the largest study to date to use MRA images to model hemodynamic behavior and wall shear stress in individuals with SCD.

## 2. Materials and Methods

To aid the reader, we have provided Supplementary Figure S1, which summarizes the overall workflow and is thus a super summary of the methodology for this study. Furthermore, because this is no longer considered human subject research, a prior IRB approval was not required.

### 2.1. SIT Trial Overview

The SIT trial was a multicenter randomized clinical trial to determine whether chronic RBC transfusion was efficacious at preventing the development of new SCI and/or progression of existing SCI over a three-year period in children with SCD and TCD velocities of <200 cm/s [21,23]. Briefly, the study included children ages 5–15 years with SCD, specifically the higher risk genotypes of HbSS and HbSβ^0^ thalassemia, a normal or conditional screening TCD (defined as time-averaged maximum mean velocity of <200 cm/s in the anterior cerebral artery (ACA), middle cerebral artery (MCA), and internal carotid artery (ICA)), and at least one infarct-like lesion on the screening MRI scan; children with other SCD genotypes and those already on disease-modifying therapy with either hydroxyurea or chronic transfusion therapy were excluded. SCI was defined as an area of hyperintensity at least 3 mm in one dimension, visible in at least 2 planes on a FLAIR T2-weighted MRI sequence, and not associated with a clinical neurological change [21,23]. Children were first screened to ensure that they met the eligibility criteria and then they were randomized to either chronic (monthly) red blood cell (cRBC) transfusions or observation only (the standard-of-care) for 36 months; participants underwent screening MRI/MRA, MRI/MRA at baseline (i.e., prior to randomization), and MRI/MRA after 36 months of cRBC therapy or observation [23]. The SIT trial utilized a standardized MRI protocol described in Supplementary Appendices by Casella et al. [23]. By utilizing participants which were imaged on different MRI scanners, though using a standardized MRI sequencing protocol, we strove to increase the generalizability of our findings. Because the original SIT trial relied on MRI findings as a primary endpoint for the study, the imaging data had to be very consistent across MRI scanners.

### 2.2. Case Selection for This Study

We randomly selected the MRA images from 10 participants (5 in the observation arm and 5 from the transfusion arm) and included images from all three time points (screening, pre-randomization, and 36 months), when available, to perform CFD and vessel tortuosity analysis. The members of our study team (SP, EG and RB) who completed the analysis were blinded to each participant’s treatment status. We also obtained information on each participant’s age at baseline, TCD velocity recorded at each time point or at a date closest to the date of MRI/MRA acquisition (when available), presence/development of neurological events (i.e., new SCI, transient ischemic attack (TIA), or overt stroke) during the three years of study follow up.

### 2.3. 3-D Model Development

The 3-dimensional models of segments of the internal carotid artery and its main branches were generated from raw MRA data using 3D Slicer [24] and then segmented into left and right sides using Autodesk Meshmixer (Autodesk, San Rafael, CA, USA). The ICA vessel was cut immediately after (~0.25 inches) the emergence of its major branches (MCA, ACA and PCA) before its branching. The result was a stereolithographic (STL) 3D image/model of the vessel segment, which was further processed and smoothed in Fluent SpaceClaim before moving onto the CFD preprocessing. The 3D models were imported into Ansys Student (Ansys Inc., Cannonsburg, PA, USA) 2021R2 (Workbench-Fluent) for 3-dimensional time-dependent CFD analysis using laminar flow assumption. A more detailed description of the CFD processing and analysis is presented below.

### 2.4. Mesh Generation

The finite element method (FEA) was used to determine correlation between the vessel geometry and hemodynamic parameters. The inlets and outlets were defined for the 3D vessel geometry as ICA and MCA/ACA respectively. The vessel geometry was discretized into a polyhedral mesh for better gradients approximation [25]. Grid/mesh convergence study was done to make sure that the velocity results are independent of the mesh size. A table for the variation of velocity and WSS for a varied number of elements is shown in Supplementary Table S1. A wide range of number of elements, i.e., from 6500 to 18,000 was considered and the effect of elements number on velocity and wall shear convergence was analyzed. The table shows that the change in velocity and shear values was low compared to the increase in mesh element number and computational time. Thus, to keep the percent error of variation for velocity and WSS at less than 5%, the mesh was set at medium smoothing with approximately 9500 elements to optimize the simulation time while maintaining the accuracy of velocity and shear parameters.

The simulation result showed convergence as shown in the Supplementary Figure S2. The time-averaged values for wall shear and velocity were calculated once both parameters were stable, and change was less than 3%. The line graph for velocity and wall shear stress along the vessel wall for one patient (AAF) in the observation arm and one patient (HAE) in the cRBC transfusion arm is shown in Supplemental Figure S3.

### 2.5. Computational Fluid Dynamics Model

#### 2.5.1. Setup and Assumptions

The laminar transient model was chosen for flow analysis. The governing CFD model utilizes the classic Navier–Stokes equation for Newtonian fluids following the conservation of momentum and mass. The equation reads as:

$$\rho \frac{Du}{Dt}+\nabla p-\mu \nabla^{2}u=0$$

where $\mu \nabla^{2}u\;$ is the reduced form of shear stress divergence , *D/Dt* is material derivative, *u* = flow velocity, ∇ = divergence, *p* = pressure, μ = dynamic viscosity, and $\nabla^{2}\;$= Laplace operator. ∇.τ as ∇.u=0 for incompressible fluid. Blood in these simulations was treated as a Newtonian fluid with constant viscosity (*µ* = 0.03 g/(cm·s), *ρ* = 1.06 g/cm^3^). Both the artery wall and blood (1.22 g/cm^3^) were assumed healthy (i.e., physiological levels of blood cells and other blood components and no evidence of vessel wall leakage). The boundary conditions were defined with a constant inlet velocity of 130 cm/s (which is based on the lower limit of the normal reported by Adams et al.) [7,26,27], with a null pressure gauge at the outlet. The velocity along the artery walls is assumed to be zero (no-slip condition).

#### 2.5.2. Solution

The area-weighted velocity at the MCA outlet and shear throughout the vessel wall were calculated at each time step. Here, the shear stress was estimated from the following equation,

$$\tau =\mu \left(\nabla u+\nabla u^{T}\right)$$

Here, the latter term is normal components of shear stress projected as tangential form. The clinical significance of wall shear in boundary wall is well defined [19,28]. The residual error convergence levels were set to 10^−6^ for each equation. We performed laminar transient simulations of time steps of 0.001 s with the total time of 3 s..

#### 2.5.3. Result

The time averaged mean velocity (TAMV) was calculated by integrating the area under the curve (AUC) after the velocity had stabilized in the simulation, and this velocity estimate was captured in Equation (1) below. The TAMV was calculated once the solution was stabilized over the multiple time steps where the changes in velocity and wall shear stress values were less than 3% between successive trials as well as between maximum and minimum value considered. Both values in the final time step were excluded from the calculation.

(1) $$\mathrm{TAMV}=\frac{area\;under\;the\;curve\;\left(auc\right)}{total\;duration\;of\;the\;flow}\;=\frac{1}{\Delta T}\;\int_{0}^{T}f\left(x\right)d\left(x\right)$$

where AUC = $\int_{0}^{T}f\left(x\right)d\left(x\right)$. The wall shear contour plots were generated at T = 3 s in the entire vessel wall and streamline plots were generated starting from the inlet.

### 2.6. Tortuosity Index Calculation

**Figure 1:**
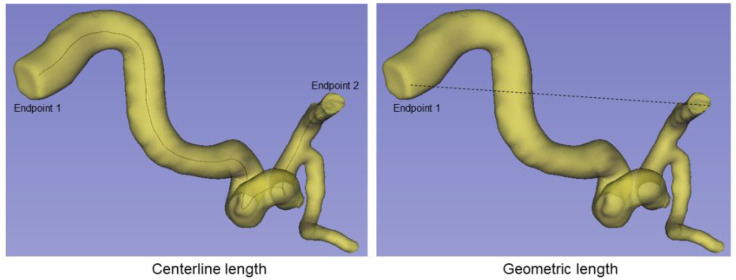
Estimation of tortuosity of vessel segment. (**Left**): Centerline represents the ICA to MCA ends. (**Right**): Geometric distance (shortest) between ICA to MCA ends.

Arterial tortuosity is defined as the measure of the convoluted pathway of blood flow within a vessel compared to the direct pathway between the two ends of the vessel and has been implicated in both genetic cardiovascular disease syndromes and in the development of white matter hyperintensities in older adults [29,30]. We calculated the tortuosity index of the vessel segment from the ICA to the MCA outlet of each 3D blood vessel model using 3D Slicer with VMTK (Vascular Modeling Toolkit). For our study, arterial tortuosity was calculated using previously published approaches [31,32,33] as the ratio of the center line length (L1) to the geometric length (L2) and then subtracting 1 (which indicates a perfect vessels with no tortuosity) from the result (Tortuosity index = $\left(L1\;÷L2\right)-1$) (see Figure 1), with larger values indicating more tortuous vessels.

### 2.7. Statistical Analysis

Statistical analysis was completed using SAS version 9.4. Demographics were compared between cRBC transfusion and observational groups. Fisher’s exact test or the chi-square test was used to analyze the categorical variables. Differences in continuous variables were evaluated using Student’s t-test and Wilcoxon rank-sum test. TAMV, tortuosity, and WSS were compared between the right and left ICA/MCA at the pre-randomization time point. TAMV at pre-randomization was then subtracted from the TAMV value at the 36-month timepoint to create change in TAMV values. Mean and standard deviation of change in TAMV values was then calculated. The mean change in TAMV was compared between the cRBC and observation groups. This process was repeated for tortuosity and WSS. The right and left ICA/MCA were analyzed separately, except when comparing left to right at the pre-randomization time point. We then performed Spearman’s correlation analysis to determine if there is a correlation between participant’s age, TCD velocity at screening and TAMV or WSS at screening or study exit. We did not examine the other time points because of the high rate of missingness for the TCD velocity data at the pre-randomization.

## 3. Results

### 3.1. Demographics

Of the ten patients included in our analysis, five had been randomized to cRBC and five to the observation group. There was no difference in age: 7.9 (1.5) vs. 7.9 (2.0) years between the participants in the cRBC arm compared to the observation arm. Each group was 20% female. At study entry the mean TCD velocity for the observation group was 136 cm/s (24.7) compared to 153 cm/s (21.8) in the cRBC group, which was not significantly different (*p* = 0.3). Among the 10 participants, there were a total of three cerebrovascular events during the 36 months of follow up: two transient ischemic attacks (one in the cRBC group and one in the observation group) and a new SCI in the observation group. None of the participants that experienced a neurological event had initial TCD velocities ≥ 200 cm/s. However, we observed that the participant who experienced a TIA on transfusion had a TCD velocity of 241 cm/s at study exit. This participant also had a TAMV of 272 cm/s on the left, based on CFD modeling from the pre-randomization MRA. Similarly, the participant in the observation arm who had TIA also had a pre-randomization TAMV of 303 cm/s on the left side; unfortunately there was no exit TCD velocity recorded for this participant. For both participants, the CFD-derived TAMV was normal on the right side and was 149 cm/s and 194 cm/s, respectively, on the left side, at study exit. The participant who had an SCI in the observation arm did not have any TAMV (from CFD modeling) or TCD velocity recording that was considered abnormal or even conditional, based on current clinical cutoffs (See Supplementary Table S1).

### 3.2. Arterial Blood Flow Velocity

**Figure 2:**
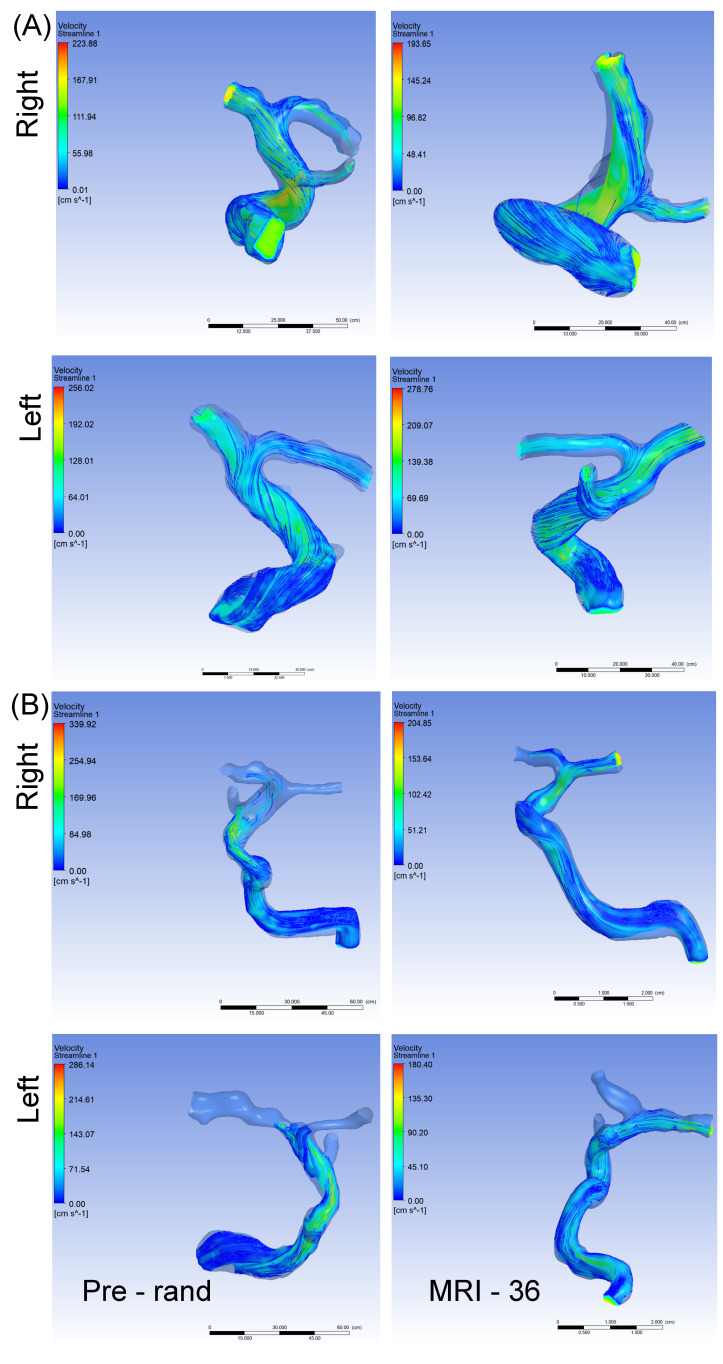
Panel “(**A**)” represents velocity in a participant in the observation arm with MRA at pre-randomization and after 36 months. In the right ICA, TAMV decreased from 168 cm/s to 125 cm/s after 36 months, while the left ICA TAMV increased from 114 cm/s pre-randomization to 166 cm/s at 36 months. Panel “(**B**)” represents velocity in a participant in the cRBC treatment arm with MRA at pre-randomization and after 36 months. In the right ICA, TAMV decreased from 186 cm/s to 154 cm/s after 36 months, while the left ICA TAMV unchanged at 102 cm/s pre-randomization and 105 cm/s at 36 months. cRBC = chronic red blood cell infusion, MRA = magnetic resonance angiography, TAMV = time averaged mean velocity.

There was no statistically significant difference between the cRBC and observation groups with regard to mean TAMV or change in TAMV from baseline, possibly due to the small sample size (Table 1). A closer look at the data (see Table 2), show a marginally significant but negative correlation (r^2^ = −0.65, *p* = 0.06) between baseline TCD velocity in all 10 participants and TAMV on the left side, at the screening time point. Similar negative correlations were observed between baseline TCD velocity and screening TAMV on the right, and baseline TCD velocity and TAMV at study exit bilaterally; however, these correlations were not statistically significant. Notably, both TCD velocity and CFD-derived TAMVs at screening were negatively correlated with participants age at baseline. When we examined the relationship between velocities and WSS, we observed a statistically significant correlation (r^2^ = 0.74, *p* = 0.04) between TAMV on the right and WSS on the right at the study exit. We also observed a similar (positive) trend in correlation between TAMV on the left and WSS on the left, as well as between TAMVs and WSS at baseline; however, these were not statistically significant (see Table 2). Figure 2A,B are representative velocity streamlines obtained from CFD simulations for participants randomized to the observation and transfusion arms, respectively. For each participant we also show simulation images from the pre-randomization (Pre-rand) and study exit (MRI-36) time points.

### 3.3. Vessel Tortuosity

Comparison of vessel tortuosity between the cRBC group and the observation group (Table 3) demonstrated no statistically significant difference in degree of ICA to MCA segment tortuosity between the groups. We observed a slight decrease in vessel tortuosity on the left among the cRBC arm, while there was a slight increase in tortuosity on the same side, for the observation arm. While these observations were not statistically significant, and we note the small sample size of this study, they demonstrate proof of principle for our methods and thus the potential when applied to a larger sample. 

### 3.4. Wall Shear Stress

**Figure 3:**
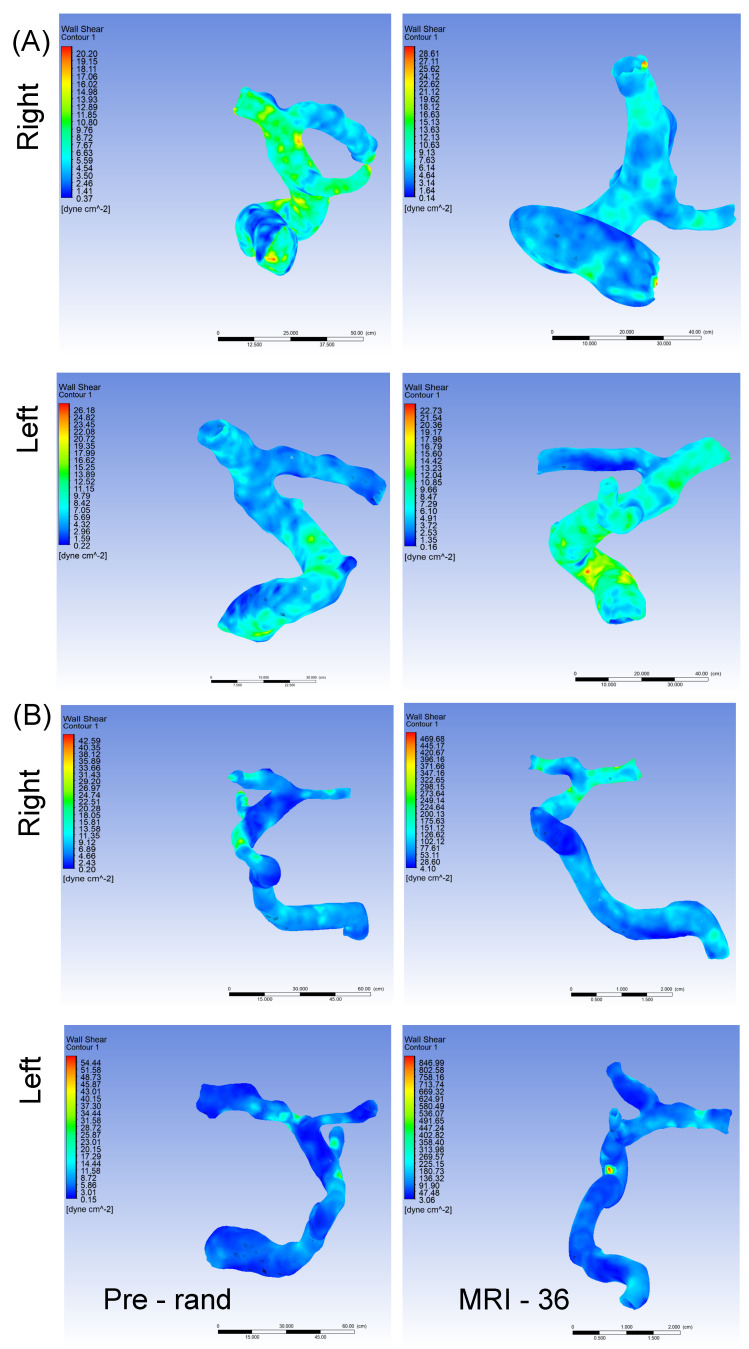
Panel “(**A**)” represents wall shear stress in a participant in the observation arm with MRA at pre-randomization and after 36 months. In the right ICA WSS further decreased from 6.9 dynes/cm^2^ pre-randomization to 5.0 dynes/cm^2^ at 36 months, while in the left ICA the WSS increased from 5.0 dynes/cm^2^ to 6.9 dynes/cm^2^. Panel “(**B**)” represents wall shear stress in a participant in the cRBC treatment arm with MRA at pre-randomization and after 36 months. In the right ICA WSS increased from 6.2 dynes/cm^2^ pre-randomization to 85.5 dynes/cm^2^ at 36 months, while in the left ICA the WSS increased from 5.3 to 88.3 dynes/cm^2^. cRBC = chronic red blood cell infusion, MRA = magnetic resonance angiography, WSS = wall shear stress.

At the screening time point there was no statistically significant correlation between WSS and either TAMV or screening TCD velocity, except for TAMV and WSS on the right. As shown in Table 4, we did not observe a statistically significant difference between the cRBC and observation group with regard to WSS at the pre-randomization time point (*p* = 0.09 and 0.11, on the right and left respectively). Additionally, at the study exit time point, the WSS was not significantly different between groups, either on the left or right side. The change from baseline was also not significantly different between groups (*p* = 0.08 and 0.30, on the right and left respectively). It is worth noting that based on currently available data, the physiologic level of WSS is 20–30 dynes/cm^2^ [34] with some studies suggesting that the lower boundary could be as low as 18 dynes/cm^2^ [35]. Low (9 dynes/cm^2^) and supra low or sub-physiologic (4.5 dynes/cm^2^) WSS levels are associated with endothelial and consequently arterial remodeling [34,35,36] and endothelial activation [37,38]. Both endothelial remodeling and activation are key components of the vascular pathobiology of SCD-related cerebrovascular complications. Thus, as shown in Table 4, at baseline, the average WSS for the cRBC arm was closer to the sub-physiologic level, while that for the observation arm was in the supraphysiologic level of ≥36 dynes/cm^2^ [35]. At study exit in Table 4 the average WSS level for the cRBC arm has increased from the sub-physiologic to the supraphysiologic levels. Unlike sub-physiologic levels of WSS, supraphysiologic levels has been reported to increase the likelihood for thrombosis [34,35]. The implication of this in SCD will become clear in larger sample analysis. Looking at Supplemental Table S2, a closer examination of the WSS data for the two participants who developed TIA indicated that the participant on cRBC had WSS values of 100.1 dynes/cm^2^ and 96.4 dynes/cm^2^ on the right and left side, respectively, at the study exit timepoint. Similarly, the participant in the observation arm who developed TIA also had WSS values that were 79.0 dynes/cm^2^ and 183.1 dynes/cm^2^ on the right and left side, respectively, at the pre-randomization time point. We did not observe elevated (>30 dynes/cm^2^) WSS values in the participant who developed SCI, however, this participant, like most in this study, had sub-physiological levels of WSS. Figure 3A,B are representative images of the distribution of WSS along the analyzed vessel segment, obtained from CFD simulations for participants randomized to the observation and transfusion arms, respectively. For each participant we also show simulation images from the pre-randomization (Pre-rand) and study exit (MRI-36) time points.

## 4. Discussion

We observed no differences in change in TAMV, WSS, and vessel tortuosity at 36 months between SCD participants treated with cRBC transfusion and those in the observation group. At baseline and 36 months our SCD participants had higher vessel tortuosity than adult healthy controls, with comparable tortuosity indices to adults with connective tissues diseases such as Marfan’s Syndrome and Loeys-Dietz Syndrome [39]. Increased vessel tortuosity has been seen in extracranial carotid and vertebral arteries in adults with SCD [40], as well as intracranially in a mouse model of SCD [41]. Similarly, the WSS was higher in our pediatric SCD participants than in healthy adult and pediatric controls in other studies using similar MRA approaches [42,43]. Our TAMV was generally higher than approaches which have used transcranial Doppler to quantify TAMV in SCD [44] which is consistent with previous findings by Rivera et al. [19]. Thus, our findings add to the literature describing intracranial vasculopathic changes resulting in greater TAMV, WSS, and vessel tortuosity in SCD. Additionally, the higher TAMV and WSS observed in our study could be attributed to the fact that we integrated our average data along the entire vessel segments (usually longer); thus, we included “hot spots” with very high local velocity and WSS, which is different from the way TCD velocity measures (TAMV) are calculated [7,45].

Among the ten participants, two in the observation group and one in the cRBC group had cerebrovascular events during the 36 months of follow up. These participants who developed cerebrovascular events had TCD velocities < 200 cm/s at baseline and pre-randomization time points. The participant on cRBC transfusion had a CFD-derived TAMV of 272 cm/s on the left at the pre-randomization time point, a TCD velocity of 241 cm/s at study exit, and WSS values of 100.1 dynes/cm^2^ and 96.4 dynes/cm^2^ on the right and left side, respectively, at the study exit timepoint. Similarly, the participant in the observation arm who developed TIA also had a CFD-derived TAMV of 303 cm/s on the left at the pre-randomization time point and WSS of 79.0 dynes/cm^2^ and 183.1 dynes/cm^2^ on the right and left side, respectively, at the pre-randomization time point. The TCD velocity value for this participant was missing for the study exit time point, while the other time points were within normal limits. Thus, based on the CFD TAMV and the WSS levels, both these participants were at increased risk for stroke as previously described [7,35,46]. While Liu et al. found that large vessel vasculopathy was associated with increased white matter disease in adults with SCD [30], our study demonstrates that cerebrovascular events occur in children with SCD with even mild large vessel vasculopathy, suggesting additional pathophysiologic mechanisms which contribute to cerebrovascular events. Certainly, large vessel vasculopathy contributes to the development of silent infarcts as well as overt cerebrovascular disease, as Guilliams et al. found a greater distribution and overall density of silent cerebral infarcts in areas with large vessel vasculopathy [47]. Low cerebral blood flow may be an additional factor resulting in cerebrovascular disease in SCD [48], particularly in the setting of large vessel vasculopathy. Likely large vessel vasculopathy interacts with a variety of factors resulting in cerebrovascular disease in SCD. One question that remains unanswered is whether the vasculopathic changes occurring in large vessels in SCD are the same as those occurring in small cerebral vessels; we hypothesis similar changes occurring in the small cerebral blood vessels contribute to SCI in SCD. However, this hypothesis has yet to be tested and we hope to do so in future studies. Furthermore, also unclear is why such dramatic changes in WSS values exist, especially in the cRBC arm. Whether this is a consistent trend is one of the questions we hope to answer with a larger sample. At the moment, we can only speculate that it might be due to a maladaptive response to changes in hemoglobin levels and other blood rheological properties. However, this will need to be supported by laboratory evidence.

Our study did not demonstrate changes in the WSS and vessel tortuosity in the cRBC transfusion group, as chronic transfusion therapy has been shown to improve vessel tortuosity in other studies and is one of the bases for the recommendation of cRBC transfusions in the setting of an abnormal TCD [7,8,46,49]. This may have been due to our small sample size. As well as preventing overt stroke, the SIT trial demonstrated a 58% relative risk reduction in the development of new SCI in those placed on the cRBC transfusion protocol [21]. In addition to the improvement in vessel tortuosity and wall shear stress, this is also likely due to an increase in both total hemoglobin and improved oxygen carrying capacity with hemoglobin A, as well as lower cerebral blood flow velocity, allowing for better perfusion of the watershed areas of the brain, which are particularly susceptible to SCI. Hydroxyurea is speculated to work in a similar way to cRBC transfusions by also increasing total hemoglobin and increasing oxygen-carrying capacity through an increase in fetal hemoglobin [50,51,52].

One obvious limitation of this study is the small samples size of ten SCD participants, which resulted in wide ranges in standard deviations, limiting our ability to detect true differences where they may exist. However, as stated earlier, this study is a demonstration of the feasibility of our model, with the plan to increase the sample size in future studies/analysis. Another limitation is non-uniform availability of hematological and other clinical measures such as blood oxygen levels. It is difficult to infer random missingness due to small sample size. Additionally, geometries were rigid, and we did not have patient-specific boundary conditions. Furthermore, viscosity was assumed to be constant and akin to literature values. Blood is a non-Newtonian fluid. Our simulations were for the large arteries, not the smaller vessels, where shear rates are lower and produce differences in viscosity between Newtonian and non-Newtonian models. An additional limitation was the availability of TCD data. The SIT trial concluded in 2014 and unfortunately TCD TAMV were not recorded for each vessel (MCA, ACA, ACA, PCA, and basilar), and only the highest TAMV was recorded for the participant at a given time point (screening, pre-randomization, and at 36 months). Therefore, we utilized these data since they reflected the highest TAMV at a given time point. Another limitation is the focus on the ICA and its branches, without analysis of the PCA and their branches. Evaluations of intracranial stenosis and tortuosity in SCD have primarily focused on the ICA/MCA junction using direct cerebral angiography and TCD [8,53,54]. As most cerebral infarcts in SCD occur in the ICA and MCA distribution, we prioritized analysis of the ICAs over the posterior cerebral arteries in this proof of principle work.

## 5. Conclusion

In this study, we demonstrated that computational fluid dynamics modelling can be applied to real-world magnetic resonance angiography imaging to determine blood flow velocities and wall shear stress, particularly in indiviudals with SCD. Although small, this first proof-of-principle study has garnered valuable insight into the pathophysiology of SCD-related large vessel vasculopathy. Future studies will involve a large sample size with the goal to better define the vascular changes that predict cognitive impairment in individals with sickle cell disease. 

## Figures and Tables

**Table 1:** Comparison of changes in time averaged mean velocity (TAMV) in observation and chronic RBC transfusion arms.

		Chronic Transfusion Therapy (*n* = 5)		Observation Only (*n* = 5)		*p*-Value
		TAMV in cm/s (SD)	Mean Change in TAMV in cm/s (SD)	TAMV in cm/s (SD)	Mean Change in TAMV in cm/s (SD)	
Pre-randomization	right	159 (26)	-	145 (42)	-	0.14 ^+^
	left	183 (65)	-	134 (68)	-	0.78 ^+^
Study exit (36 months)	right	165 (60)	6 (59)	137 (47)	−8 (69)	0.74 ^++^
	left	168 (67)	−0.15 (101)	197 (67)	62 (97)	0.25 ^++^

cRBC = chronic blood transfusion, cm/s = centimeters per second, SD = standard deviation. ^+^ = compared to observational group pre-randomization, ^++^ = compared to change in observational group.

**Table 2:** Showing results of Spearman’s correlation analysis, to determine whether there is a correlation between participant’s age, TCD velocity at screening and TAMV or WSS at screening or study exit. We did not examine the other time points because of high rate of missingness for the TCD velocity data at the pre-randomization and study exit time points.

	TAMV (cm/s) Right Screening (*p*-Value)	TAMV (cm/s) Left Screening (*p*-Value)	TAMV cm/s Right Study Exit (36 Months) (*p*-Value)	TAMV cm/s Left Study Exit (36 Months) (*p*-Value)	TCD Velocity (cm/s) Study Entry (*p*-Value)
Age	−0.13 (0.73)	0.27 (0.45)	−0.31 (0.46)	−0.17 (0.69)	-
WSS Right Screening	0.38 (0.28)	−0.20 (0.58)	-	-	0.25 (0.52)
WSS Left Screening	0.38 (0.28)	0.38 (0.28)	-	-	−0.17 (0.67)
WSS Right Study exit	-	-	0.74 (0.04)	0.50 (0.21)	0.12 (0.78)
WSS Left Study exit	-	-	0.55 (0.16)	0.26 (0.53)	0.33 (0.42)
TCD Velocity Study Entry	−0.40 (0.29)	−0.65 (0.06)	−0.02 (0.96)	−0.57 (0.14)	-

*p*-value = two tailed *p*-value, TAMV = timed average mean velocity, TCD = transcranial Doppler, WSS = wall shear stress.

**Table 3:** Comparison of changes in tortuosity in observation and chronic RBC transfusion arms.

		Chronic Transfusion Therapy (*n* = 5)		Observation Only (*n* = 5)		*p*-Value
		Tortuosity Index Mean (SD)	Mean Change in Tortuosity Index (SD)	Tortuosity Index Mean (SD)	Mean Change in Tortuosity Index (SD)	
Pre-randomization	right	0.85 (0.45)		0.90 (0.30)		0.84 ^+^
	left	0.87 (0.46)		0.60 (0.17)		0.25 ^+^
36 Month	right	0.99 (0.18)	0.15 (0.30)	0.92 (0.42)	0.02 (0.27)	0.49 ^++^
	left	0.82 (0.18)	−0.04 (0.34)	0.70 (0.26)	0.10 (0.21)	0.46 ^++^

^+^ = two tailed *p*-value for pre-randomization tortuosity in the observation group vs. the cRBC group. ^++^ = two tailed *p*-value for mean change in tortuosity in the observation group vs. the cRBC group. cRBC = chronic blood transfusion, SD = standard deviation.

**Table 4:** Comparison of changes in wall shear stress (WSS) in observation and chronic RBC transfusion arms.

		Chronic Transfusion Therapy (*n* = 5)		Observation Only (*n* = 5)		*p*-Value
		WSS Mean in Dyne/cm^2^ (SD)	Mean Change in WSS (SD)	WSS Mean in Dyne/cm^2^ (SD)	Mean Change in WSS (SD)	
Pre-randomization	right	5.64 (1.44)		36.27 (36.13)		0.09 ^+^
	left	6.25 (1.68)		57.03 (62.6)		0.11 ^+^
36 Month	right	61.85 (45.82)	56.20 (44.73)	41.03 (42.69)	4.76 (35.02)	0.08 ^++^
	left	65.87 (49.26)	59.62 (49.70)	70.23 (50.11)	13.20 (79.69)	0.30 ^++^

^+^ = two tailed *p*-value for pre-randomization WSS in the observation group vs. the cRBC group. ^++^ = two tailed *p*-value for mean change in WSS in the observation group vs. the cRBC group. cRBC = chronic blood transfusion, SD = standard deviation.

## Data Availability

The data included in this study are available from the corresponding author upon request and processing of required DUA/MTA.

## Supplementary Materials

The following supporting information can be downloaded at: https://www.mdpi.com/article/10.3390/brainsci12101402/s1, Figure S1: Study framework/workflow; Figure S2: Simulation convergence results; Figure S3: The line graph for velocity and wall shear stress along the vessel wall for one patient (AAF) in the observation arm and one patient (HAE) in the cRBC transfusion arm. Table S1: Mesh convergence study showing percentage variation in velocity and wall shear with respect to baseline value (9500 elements); Table S2: Presentation of some relevant individual characteristics for each participant.
