# Supplementary material for: Effect of Blood Transfusion on Cerebral Hemodynamics and Vascular Topology Described by Computational Fluid Dynamics in Sickle Cell Disease Patients
Source: Brain Sci. 2022 Oct 18;12(10):1402. doi: 10.3390/brainsci12101402 (PMC9599808; doi:10.3390/brainsci12101402)
Supplement: Supplementary file 1 [file brainsci-12-01402-s001.zip › brainsci-1961077 -Table S1.pdf]

**Supplementary Table S1**. Mesh convergence study showing percentage variation in velocity and wall shear with respect to baseline value (9500 elements). **(A)** Percent error of Velocity (measured in MCA outlet) with respect to change in umber of elements. **(B)** Percent error of Wall shear (measured vessel wall) with respect to change in umber of elements.

(A)

| Total Number of Mesh elements | Absolute Percentage Variation of velocity |
|---|---|
| 6500 | 0.35 |
| 10000 | 0.52 |
| 12000 | 3.15 |
| 15500 | 4.02 |
| 18000 | 4.89 |

(B)

| Total Number of Mesh elements | Absolute Percentage Variation of Wall Stress |
|---|---|
| 6500 | 3.70 |
| 10000 | 1.85 |
| 12000 | 0.74 |
| 15500 | 7.40 |
| 18000 | 9.07 |
