# Supplementary material for: Effect of Blood Transfusion on Cerebral Hemodynamics and Vascular Topology Described by Computational Fluid Dynamics in Sickle Cell Disease Patients
Source: Brain Sci. 2022 Oct 18;12(10):1402. doi: 10.3390/brainsci12101402 (PMC9599808; doi:10.3390/brainsci12101402)

Supplementary Figure S1

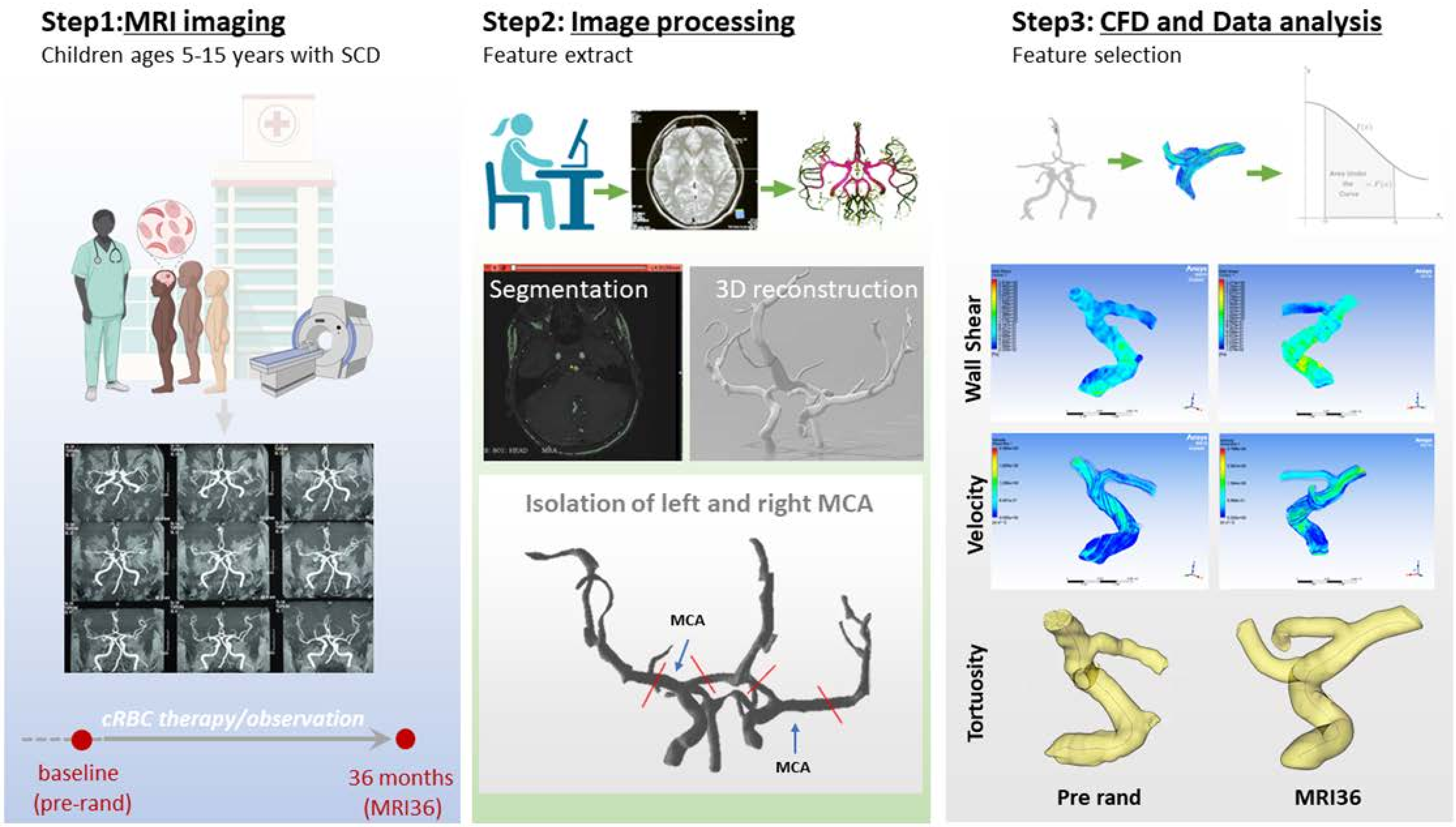

Supplement: Supplementary file 1 [file brainsci-12-01402-s001.zip › brainsci-1961077- Figure S1.pdf]
