# Supplementary material for: Effect of Blood Transfusion on Cerebral Hemodynamics and Vascular Topology Described by Computational Fluid Dynamics in Sickle Cell Disease Patients
Source: Brain Sci. 2022 Oct 18;12(10):1402. doi: 10.3390/brainsci12101402 (PMC9599808; doi:10.3390/brainsci12101402)
Supplement: Supplementary file 1 [file brainsci-12-01402-s001.zip › brainsci-1961077- Figure S3.pdf]

(A) AAF MRI36 R

(B) HAE MRI36 R

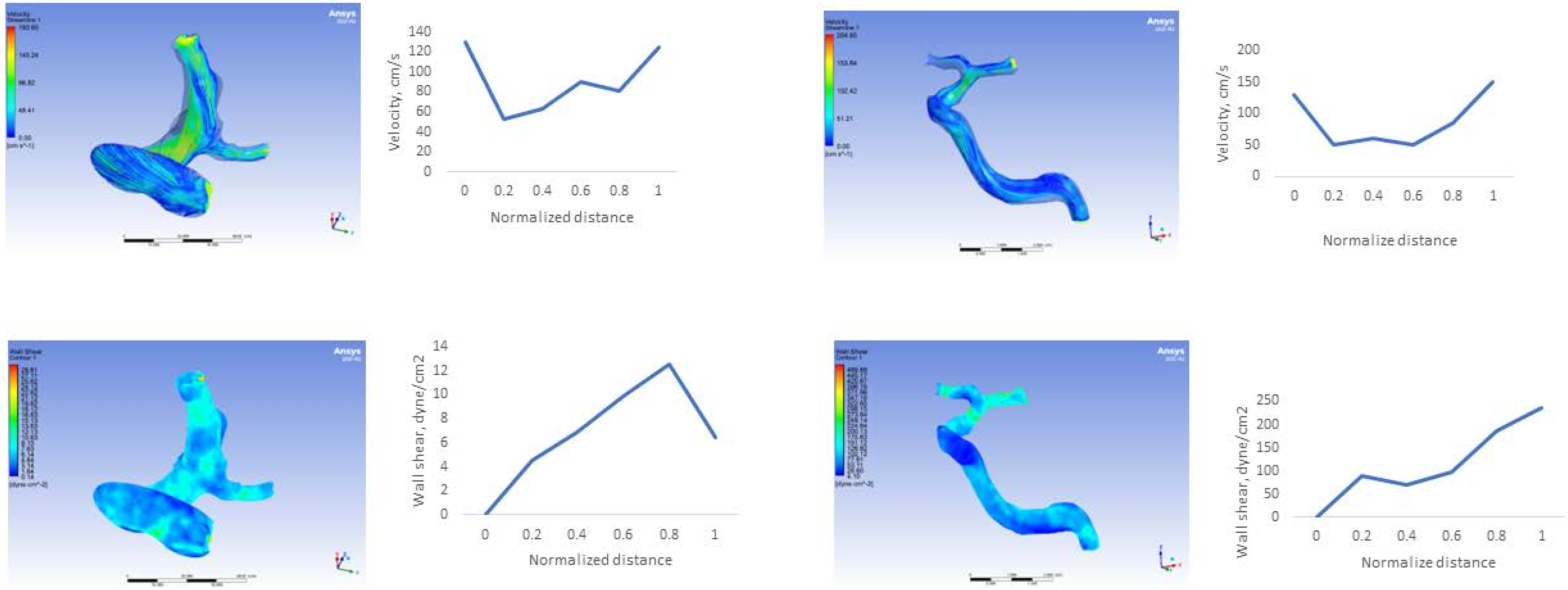

Supplementary Figure S3. The line graph for velocity and wall shear stress along the vessel wall for one patient (AAF) in the observation arm and one patient (HAE) in the cRBC transfusion arm. In both cases, images from the study exit (MRI36) time point was used
